# Preliminary evidence for reduced adipose tissue inflammation in vegetarians compared with omnivores

**DOI:** 10.1186/s12937-019-0470-2

**Published:** 2019-08-12

**Authors:** Maria E. Morgan-Bathke, Michael D. Jensen

**Affiliations:** 1 Endocrine Research Unit, Mayo Clinic, 200 1st St SW, Rm 5-194 Joseph, Rochester, MN 55905 USA; 2 Nutrition and Dietetics Department, Viterbo University, La Crosse, WI USA

**Keywords:** Adipocytes, Macrophages, Obesity, Fatty acids, Intramyocellular ceramides, TNF

## Abstract

**Background:**

There are links between obesity and inflammation that may relate activation of pro-inflammatory pathways by dietary factors. Because dietary fat intake of vegetarians is thought to be more beneficial than that of omnivores, we hypothesized that obese vegetarians would have less adipose tissue inflammation and lower intramyocellular ceramide concentrations than equally obese omnivores.

**Methods:**

Eight obese vegetarian (1 male) and 8 obese omnivore volunteers (1 male) completed a Food Frequency Questionnaire, underwent body composition measures, subcutaneous adipose tissue and muscle biopsies. We used immunohistochemistry to measure adipose macrophage (ATM) and senescent cells. Plasma free fatty acid (FFA), adipose FA and muscle ceramide profiles were measured using liquid chromatography/mass spectrometry. Student *t* tests were used for the comparison of primary outcomes; univariate regression analysis was used to test for associations between dietary patterns and ATMs (secondary analysis).

**Results:**

There were no differences in age (38 ± 8 vs. 39 ± 8 years), BMI (32.2 ± 2.6 vs. 33.3 ± 1.9 kg/m^2^) or percent body fat (44 ± 8 vs. 45 ± 4) between the vegetarians and omnivores. Vegetarians consumed 42% (*P* = 0.02) less saturated fat and 50% (*P* = 0.04) less cholesterol than the omnivores. Plasma FFA of vegetarians had lesser proportions of palmitic acid (24 ± 3 vs. 29 ± 4%, *P* = 0.02) and vegetarians had fewer femoral pro-inflammatory ATMs than omnivores (3.6 ± 2.8 vs. 7.9 ± 4.4 per 100 adipocytes, respectively; *P* = 0.02). Omnivores had 50% greater (*P* = 0.01) expression of *TNF* mRNA in abdominal fat. We found no significant between group differences in muscle ceramide concentrations.

**Conclusions:**

Although the sample size is small, these results may indicate that dietary patterns play a role in adipose tissue inflammation, as reflected by reduced number of femoral ATMs in obese vegetarians than obese omnivores.

## Background

Although obesity is associated with numerous co-morbidities, the underlying mechanism(s) for these metabolic abnormalities is not yet clear. Recent studies suggest a link between adipose tissue inflammation and the chronic diseases associated with obesity [1–3]. Adipose tissue macrophages (ATMs), and especially pro-inflammatory, or M1 ATMs, are particularly associated with insulin resistance (reviewed in [4]). Whether the accumulation of ATMs in humans is strictly related to fat gain or may be influenced by diet is unknown.

Vegetarian diets emphasize vegetables, legumes, and fruits while excluding meats (although variations of vegetarianism may allow some forms of seafood) and therefore contain less saturated fat. Vegetarian diets have been shown to improve blood lipids, glycemic control and blood pressure [5] and are associated with reduced inflammation [6]. Ceramides have been linked to inflammation and insulin resistance in muscle cell models [7] and exposure to saturated fatty acids has been shown to increase muscle ceramides in rodents [8]. Ceramides have also been linked to adverse muscle profiles in humans [9]. We hypothesized that the proposed benefits of a vegetarian diet would be especially notable in obese adults in that they would have less adipose inflammation and reduced muscle ceramide content than equally obese omnivores.

## Methods

### Study protocol

The participant characteristics are outlined in Table 1. The study cohort included 16 participants, 8 vegetarians and 8 omnivores, participants were matched for age and BMI. We excluded participants with chronic conditions such as diabetes or cardiovascular disease. All vegetarian participants reported following a vegetarian or vegan diet for at least 5 years prior to the study. Two vegans, one lacto-ovo vegetarian, one pescatarian, and four lacto-vegetarians volunteered for this study.

**Table 1:** Subject Characteristics

	Vegetarian (*n* = 8, Male = 1)	Omnivore (*n* = 8, Male =1)	*P*-Value
Age (years)	38 ± 8	39 ± 8	0.96
BMI (kg/m^2^)	32.2 ± 2.6	33.3 ± 1.9	0.33
Abdominal adipocyte size (μg lipid/cell)	0.72 ± 0.31	0.91 ± 0.37	0.25
Femoral adipocyte size (μg lipid/cell)	0.99 ± 0.43	1.16 ± 0.41	0.27
Total body fat (kg)	37.1 ± 7.9	41.3 ± 7.8	0.30
Fat free mass (kg)	48.7 ± 9.3	51.9 ± 4.9	0.41
Visceral Fat (kg)	4.7 ± 2.2	3.2 ± 1.3	0.12
Waist to Hip Ratio	0.87 ± 0.08	0.86 ± 0.04	0.84
Fasting blood glucose (mg/dL)	89 ± 13	91 ± 13	0.82
Systolic Blood Pressure	114 ± 15	123 ± 19	0.31
Diastolic Blood Pressure	67 ± 14	76 ± 12	0.25

Data are shown as means ± SD; *n*, number of subjects. BMI, body mass index. Visceral fat data for vegetarians n = 7

After obtaining informed consent a fasting blood sample was collected. The blood sample for one of the vegetarian volunteers was mishandled and thus we have plasma concentration data for only 7 vegetarians (Male = 1). All participants completed a Food Frequency Questionnaire using the VioScreen (Princeton, NJ) web-based software program and underwent a DEXA scan (Lunar iDXA, GE Healthcare, Madison, WI) and an abdominal single slice CT to assess body composition. One vegetarian volunteer did not have the CT scan. Visceral fat mass was measured using a single-slice abdominal computed tomography scan at the L_2–3_ level combined with DXA-measured total abdominal fat content [10]. Subcutaneous abdominal (lateral to the umbilicus) and femoral (on the anterior-lateral aspect of the mid-thigh) adipose tissue biopsies were collected using a needle liposuction technique under sterile conditions using local anesthesia. Immediately thereafter a muscle biopsy from the vastus lateralis was collected using the same sterile precautions and local anesthesia.

### Muscle tissue collection and analysis

Muscle tissue (∼300 mg) collected from biopsies was immediately processed and extractions were completed for sphingolipids concentrations as previously described [11].

### Food frequency questionnaire (FFQ)

All participants completed the VioScreen FFQ under the supervision of a Registered Dietitian. VioScreen is a validated, online food recall method to assess an individual’s mean dietary intake for 1–3 months.

### Blood collections

A Beckman Instrument (Fullerton, CA) was used to measure plasma glucose. Plasma total FFA concentrations were measured using LC/MS [12]. Plasma IL-6 and TNF concentrations were measured in 10 of the volunteers (*n* = 5 omnivores) using the Meso Scale Discovery (MSD) pro-inflammatory Panel 1 assay (cat #K15049D) in Immunochemical Core Laboratory of Mayo Clinic as described by the manufacturer. Reportable ranges were 1.58–488 pg/mL for IL-6 and 0.69–248 pg/mL for TNF.

### Adipose tissue analyses

Fat cell size was measured as previously described [13]. The composition of fatty acids in tissue lipids was assessed by extracting tissue lipids using the Folch method [14]. The samples were processed as previously described for analysis by LC/MS [12, 15]

For immunohistochemistry (IHC), adipose tissue samples (~ 350-400 mg) were processed and the staining procedure was performed as previously described [16]. The adipose tissue sections were visualized by light microscopy using an Olympus BX43 microscope. Ten random images per slide were taken at 40x magnification and two independent observers counted positively stained macrophages, crown-like structures (CLS) and total adipocytes for each field of view. Data are expressed as number of positive cells per 100 adipocytes.

To measure the senescent cell burden of adipose tissue we stained for the percentage of positive cells for senescence-associated β-galactosidase activity [17].

### Real time PCR

We were able to isolate RNA from abdominal and femoral adipose samples for all omnivores and 5 of the 8 vegetarians using the RNeasy Lipid Tissue mini kit (Qiagen # 74804). The isolated RNA was then reverse transcribed using the High Capacity cDNA Reverse Transcription kit (Applied Biosystems #4368813) as described by the manufacturer into cDNA. RT-PCR was performed using Taqman Gene Expression assays (Applied Biosystems (*IL-6* = Hs00174131_m1, *TNF* = Hs00174128_m1 and CYCA = Hs99999904_m1) and TaqMan Fast Advanced Master Mix (Applied Biosystems #4444964) on an ABI Quant thermocycler using “Fast” settings in duplicate. The ΔΔCt method was used to analyze the data. CYCA was used to normalize samples.

### Calculations and statistical methods

Values are provided as the mean ± SD when describing groups and mean ± SEM when presenting between-group comparisons. The normality of variables was determined using the Shapiro-Wilk test. IHC data are expressed as number of positive cells per 100 adipocytes, however, because adipocyte size may vary between adipose regions and individuals, we also report ATM relative to tissue mass (per gram tissue) as previously described [16]. Senescent cell data is presented per 100 nuclei. Plasma FFA and adipose tissue fatty acid composition are reported as the percent of total fatty acids. Muscle ceramide values are expressed as pmol per mg of protein.

We found no publications that provide data regarding the specific adipose and muscle research outcomes that we planned to undertake in vegetarians and omnivores. However, other studies have compared the inflammatory status of obese adults utilizing ELISAs for plasma TNF-α and IL-6 concentrations*,* two of the methods we employed. To prospectively gain some estimate for statistical power, we used the average of the mean and standard deviation values from these studies. [18–20] (TNF-α and IL-6 of 4.33 ± 0.73 pg/mL and 3.6 ± 0.43 pg/mL, respectively); our calculation indicated that to detect 50% lesser (one-sided test) TNF-α or IL-6 concentrations in vegetarians compared with omnivores with 80% power at a *P* < 0.05 would require 5 participants in each group.

Our primary hypotheses were related to the adipose tissue inflammatory status, plasma fatty acids, dietary intake, body composition and fatty acid composition of adipose tissue and muscle differences between omnivores and vegetarians. Non-paired, two tailed Student *t* tests were used for the comparison of primary analysis results between the omnivore and vegetarian groups with a *P* value of 0.05. We hypothesized that omnivores would have greater adipose tissue inflammatory status as well as greater saturated plasma and adipose tissue fatty acids when compared to the vegetarian group. Our secondary analysis evaluated the Pearson correlation between dietary intake and adipose tissue inflammation utilizing a univariate regression analysis. We hypothesized that dietary factors known to be detrimental to metabolic health would be positively correlated to adipose tissue macrophage burden. For these secondary analyses calculations a Bonferroni correction was used to reduce the likelihood of a type 1 statistical error. To compare adipose fatty acid composition between depots we used paired t-tests.

## Results

### Subject characteristics and food frequency questionnaire

Subject characteristics are provided in Table 1. The groups did not differ significantly with respect to age, BMI, body composition or blood pressure. Fasting plasma insulin concentrations 6 ± 1 and 9 ± 2 uIU/mL in vegetarians (*n* = 7, Male = 1) and omnivores (*n* = 8, Male = 1, *P =* 0.25*)*. Plasma TNF (1.8 ± 0.3 vs. 1.5 ± 0.2 pg/ml, respectively, *P* = 0.12) and IL-6 (1.0 ± 0.5 vs. 0.9 ± 0.6 pg/ml, respectively, *P* = 0.86) concentrations were not different between the 5 omnivores (Male = 1) and 5 vegetarians (Male = 1) that we had samples to analyze.

The mean reported dietary intake values are outlined in Table 2. For vegetarian participants, adherence to the vegetarian diet was confirmed via analysis of the FFQ data. We did not observe consistent differences in macro- or micronutrient intake among the vegetarian subtypes. There was no significant difference in reported total caloric intake between the omnivores and vegetarians (*P* = 0.46). The vegetarians reported significantly less saturated fat intake (*P* = 0.02), but the reported intake of monounsaturated fat (*P* = 0.27) and polyunsaturated fat (*P* = 0.46) was not statistically different between the groups. The omnivore group reported consuming greater amounts of cholesterol (*P* = 0.04) and vitamin D (*P* = 0.004), whereas vegetarians reported greater intake of fiber (*P* = 0.004) and vitamin C (*P* = 0.04).

**Table 2:** Food Frequency Questionnaire

	Vegetarian (*n* = 8)	Omnivore (*n* = 8)	*P*-Value Between Groups
Total Energy (kcal/d)	2044 ± 672	2009 ± 344	0.46
Total Fat (g/d)	67.2 ± 27.5	81.3 ± 25.2	0.18
Saturated Fat (g/d)	15.9 ± 9.9	27.6 ± 9.7	0.02
Monounsaturated Fat (g/d)	25.9 ± 10.7	29.9 ± 9.8	0.27
Polyunsaturated Fat (g/d)	16.7 ± 4.2	16.4 ± 5.1	0.46
Cholesterol (mg/d)	175 ± 152	348 ± 286	0.04
Trans Fat (g/d)	2.3 ± 1.7	2.9 ± 0.9	0.23
Carbohydrates (g/d)	288 ± 101	272 ± 49	0.36
Protein (g/d)	82.9 ± 23.4	92.7 ± 18.6	0.14
Fruit Servings/d	2.6 ± 1.7	2.0 ± 1.3	0.24
Vegetable Servings/d	6.9 ± 2.8	5.3 ± 3.2	0.09
Whole Grain Servings/d	3.7 ± 2.9	1.7 ± 1.1	0.05
Added Sugar (g/d)	39.2 ± 13.2	58.8 ± 12.8	0.14
Alpha-tocopherol (mg/d)	13.9 ± 4.5	12.0 ± 5.5	0.25
Fiber (g/d)	34.7 ± 10.5	21.4 ± 6.7	0.004
Omega-3 (g/d)	1.6 ± 0.5	1.8 ± 0.7	0.32
Selenium (mg/d)	0.13 ± 0.19	0.13 ± 0.10	0.48
Vitamin C (mg/d)	173 ± 82.7	105 ± 58.0	0.04
Vitamin D (IU/d)	136 ± 200	324 ± 52	0.004

Data are shown as means ± SD. Kcal – kilocalorie; g – gram; mg – milligram; d – day

### Adipose tissue fatty acid composition (Table 3)

Vegetarians had a significantly greater proportion of oleate in the abdominal depot than the omnivores. The vegetarians also had greater proportions of oleic, palmitelaidic, linolenic and myristic fatty acids in the femoral depot. When considering data from all subjects combined, there were greater proportions of linoleate and stearate in the abdominal depot and greater proportions of oleate in the femoral depot.

**Table 3:** Adipose Tissue Fatty Acid Composition

Fatty Acid	Abdominal			Femoral			Depots		
	Vegetarian (*n* = 8)	Omnivore (*n* = 8)	*P*-Value	Vegetarian (*n* = 8)	Omnivore (*n* = 8)	*P*-Value	Abdominal (*n* = 16)	Femoral (*n* = 16)	*P*-Value
Arachidonic	0.45 ± 0.15	0.57 ± 0.09	0.07	0.81 ± 0.42	0.41 ± 0.3	0.07	0.51 ± 0.14	0.61 ± 0.41	0.46
DHA	0.10 ± 0.06	0.12 ± 0.03	0.25	0.08 ± 0.03	0.14 ± 0.04	0.06	0.11 ± 0.05	0.1 ± 0.04	0.91
Eladic	1.7 ± 0.5	2.1 ± 0.6	0.07	3.3 ± 2.8	1.3 ± 0.9	0.08	1.9 ± 0.6	2.3 ± 2.3	0.52
EPA	0.04 ± 0.02	0.04 ± 0.01	0.33	0.1 ± 0.1	0.07 ± 0.05	0.21	0.04 ± 0.02	0.09 ± 0.08	0.05
Linoleic	23.2 ± 3.9	21.7 ± 2.4	0.22	14.2 ± 11.4	13.8 ± 10.8	0.36	22.4 ± 3.2	14.0 ± 10.7	0.01
Linolenic	1.0 ± 0.2	0.8 ± 0.2	0.08	1.5 ± 0.9	0.8 ± 0.1	0.02	0.9 ± 0.2	1.1 ± 0.7	0.19
Myristic	2.5 ± 0.8	2.5 ± 0.7	0.49	2.8 ± 1.4	2.0 ± 0.5	0.03	2.5 ± 0.7	2.4 ± 1.1	0.95
Oleic	40.0 ± 3.0	38.0 ± 2.4	0.01	46.9 ± 5.6	40.0 ± 2.8	0.01	39.1 ± 2.8	43.4 ± 5.5	0.008
Palmiteladic	0.2 ± 0.1	0.2 ± 0.1	0.24	0.2 ± 0.1	0.2 ± 0.1	0.02	0.2 ± 0.1	0.21 ± 0.1	0.19
Palmitic	23.0 ± 2.2	24.8 ± 1.0	0.05	23.9 ± 4.5	22.2 ± 2.2	0.2	23.9 ± 1.9	23.0 ± 3.5	0.38
Palmitoleic	3.2 ± 1.2	3.6 ± 0.8	0.25	4.1 ± 1.7	4.1 ± 2.4	0.5	3.4 ± 1.0	4.1 ± 2.0	0.17
Stearic	4.7 ± 1.3	5.5 ± 1.5	0.19	3.3 ± 1.0	3.6 ± 1.5	0.28	5.1 ± 1.4	3.5 ± 1.3	0.01

All fatty acids are given as the percent of total fatty acids. Data are shown as means ± SD

DHA - docosahexaenoic acid; EPA - eicosapentaenoic acid; FA – fatty acids

### Plasma free fatty acids

Plasma FFA palmitate was a lesser percent of total FFA in vegetarians than omnivores (Table 4), consistent with the greater saturated fatty acid intake reported by omnivores. There was no significant difference between the vegetarian and omnivore groups for the other fatty acid species.

**Table 4:** Plasma Fatty Acid Composition

Fatty Acid	Vegetarian	Omnivore	*P*-Value Between Groups
Arachidonic	1.0 ± 0.4	1.0 ± 0.3	0.39
DHA	0.6 ± 0.2	0.9 ± 0.5	0.09
Eladic	2 ± 0.5	3 ± 0.6	0.19
EPA	0.5 ± 0.3	0.4 ± 0.2	0.49
Linoleic	23 ± 3	21 ± 3	0.12
Linolenic	3 ± 0.4	3 ± 0.5	0.09
Myristic	2 ± 0.6	2 ± 0.4	0.22
Oleic	33 ± 3	30 ± 3	0.10
Palmitoleic + Palmiteladic	2 ± 0.6	2 ± 0.5	0.7
Palmitic	24 ± 3	28 ± 4	0.02
Stearic	8 ± 3	9 ± 2	0.34

All fatty acids are given as the percent of total fatty acids. Data are shown as means ± SD and represent the percentage of total plasma fatty acids

DHA - docosahexaenoic acid; EPA - eicosapentaenoic acid

### Adipose tissue inflammation

**Fig. 1:**
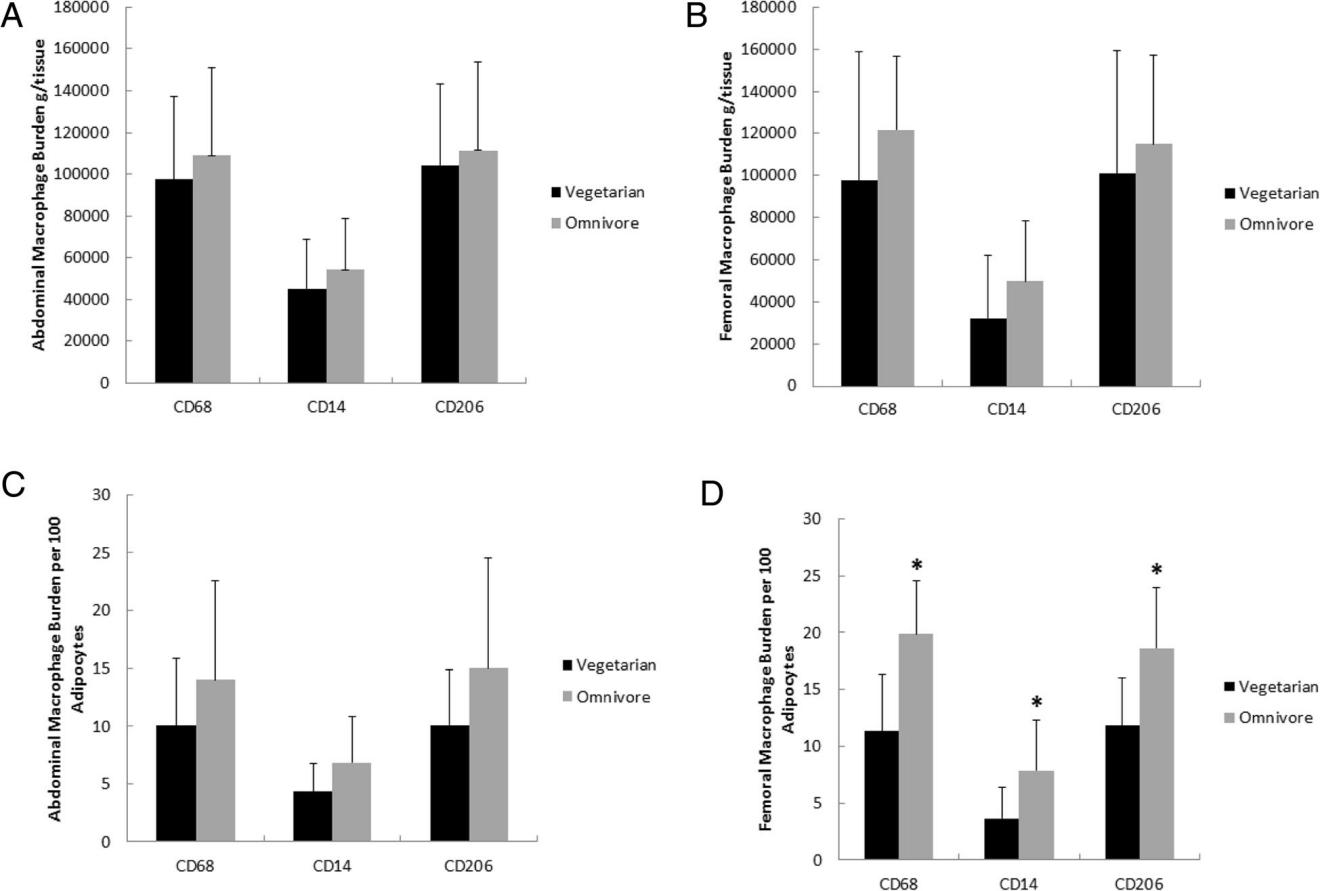
Variations in macrophage burden between groups. Significant differences (*p* < 0.05) were determined using a student’s t-test. Asterisks above groups are used to signify statistical significance. Data are presented as the mean ± SD. **a**. Abdominal macrophage burden per g/tissue. **b**. Femoral macrophage burden per g/tissue. **c**. Abdominal macrophage burden per 100 adipocytes. **d**. Femoral macrophage burden per 100 adipocytes

**Fig. 2:**
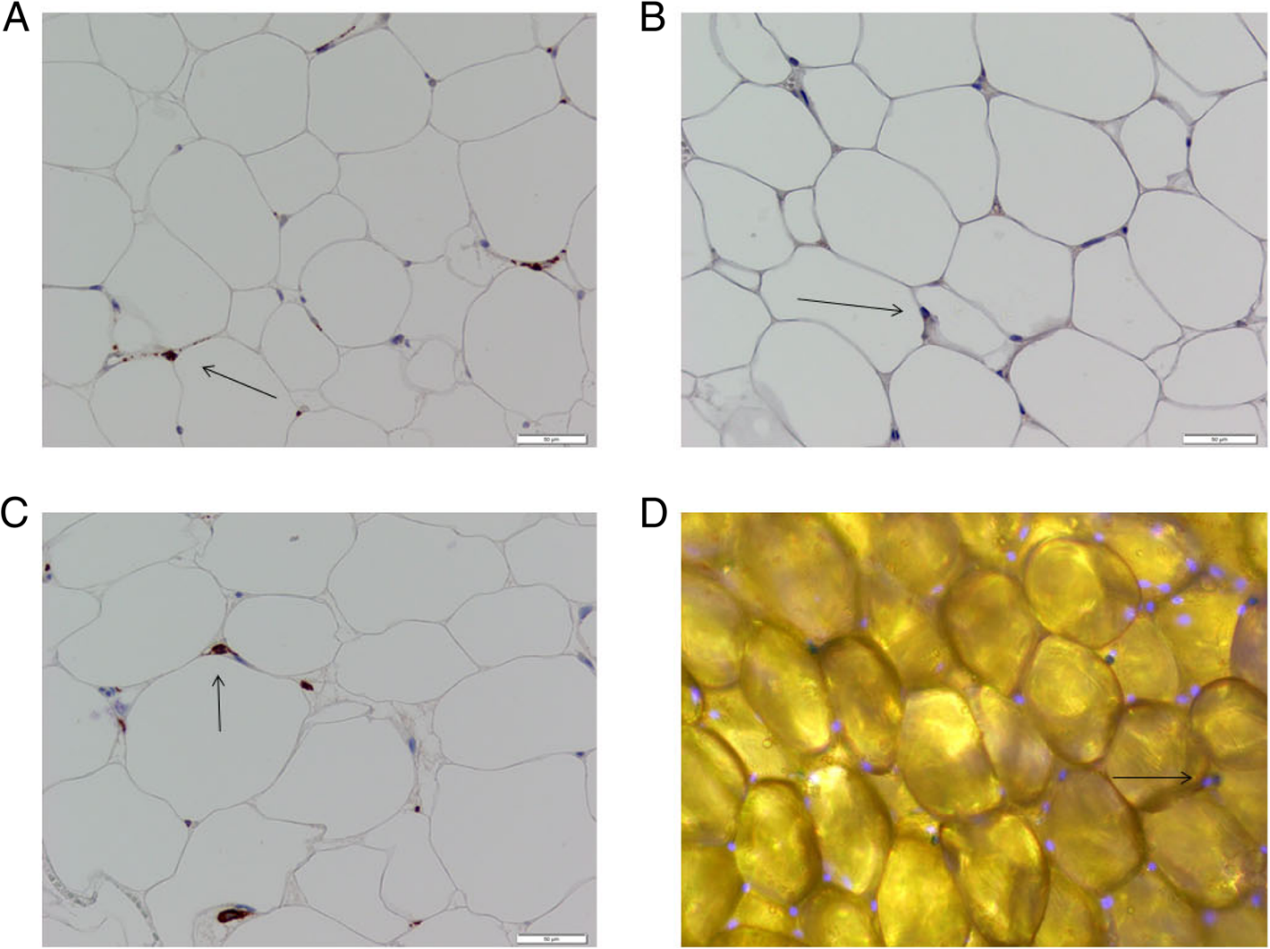
Representative images of IHC staining. Positively stained cells indicated with arrows. **a**. CD68 staining. **b**. CD14 staining. **c**. CD206 staining. **d**. SA-βgal

There was no significant difference between the groups in abdominal or femoral adipose tissue depot macrophage burdens when expressed per gram of tissue (Fig. 1a-b and Table 5, representative images Fig. 2**)**.

**Table 5:** Adipose Tissue Macrophage Burden

Macrophage	Abdominal			Femoral		
	Vegetarian (*n* = 8)	Omnivore (*n* = 8)	*P*-Value	Vegetarian (*n* = 8)	Omnivore (*n* = 8)	*P*-Value
CD68 (g/tissue)	97,540 ± 39,887	108,913 ± 42,050	0.59	97,542 ± 61,479	121,874 ± 34,699	0.17
CD14 (g/tissue)	45,196 ± 23,459	54,319 ± 24,628	0.46	32,294 ± 29,735	49,726 ± 28,638	0.12
CD206 (g/tissue)	104,244 ± 38,893	111,386 ± 42,418	0.73	100,792 ± 58,662	115,047 ± 42,110	0.29
CD68 (per 100 adipocytes)	10.0 ± 5.8	14.0 ± 8.6	0.31	11.3 ± 5.0	19.9 ± 4.7	0.001
CD14 (per 100 adipocytes)	4.3 ± 2.4	6.8 ± 4.0	0.15	3.6 ± 2.8	7.9 ± 4.4	0.02
CD206 (per 100 adipocytes)	10.1 ± 4.7	15.0 ± 9.5	0.21	11.9 ± 4.2	18.6 ± 5.3	0.01

Data are shown as means ± SD

Likewise, the number of abdominal adipose tissue macrophages was not different when expressed per 100 adipocytes (Fig. 1c and Table 5**)**. However, vegetarians had fewer femoral CD68 macrophages than omnivores (11.3 ± 5.0 v. 19.9 ± 4.7 per 100 adipocytes, respectively, *P =* 0.001). In addition, the CD14 macrophage content of femoral adipose tissue was less in vegetarians than the omnivores (3.6 ± 2.8 vs. 7.9 ± 4.4 per 100 adipocytes, respectively, *P* = 0.02). Omnivores had more CD206 macrophages in femoral fat than vegetarians (18.6 ± 5.3 vs. 11.9 ± 4.2 per 100 adipocytes, respectively, *P* = 0.01) (Fig. 1d and Table 5**)**.

There was no significant difference in adipose tissue senescent cell content between vegetarians and omnivores in abdominal (3 ± 2 vs 4 ± 3 per 100 cells respectively, *P* = 0.14) or femoral (5 ± 2 vs. 4 ± 3 per 100 cells, respectively, *P* = 0.06) fat (Fig. 2).

Abdominal adipose *TNF* mRNA expression was greater in omnivores than vegetarians (0.81 ± 0.28 vs. 0.44 ± 0.2, respectively, *P* = 0.01), whereas the differences in *IL-6* mRNA expression did not reach statistical significance (0.07 ± 0.06 vs. 0.02 ± 0.02, respectively, *P* = 0.06). There were no significant differences in femoral adipose tissue expression of either *TNF* mRNA (0.74 ± 0.27 vs. 0.58 ± 0.12, respectively, *P* = 0.17) or *IL-6* mRNA (0.03 ± 0.05 vs. 0.03 ± 0.04, *P* = 0.83) between omnivores and vegetarians.

### Muscle ceramide content

There were no significant differences between the two groups in muscle ceramide concentrations (Table 6).

**Table 6:** Muscle Ceramide Concentration

	Vegetarians	Omnivores	*P*-value
C14-Cer pmol/mg protein	28 ± 13	31 ± 13	0.33
C16-Cer pmol/mg protein	115 ± 44	108 ± 17	0.33
C18-Cer pmol/mg protein	148 ± 64	141 ± 39	0.38
C18:1-Cer pmol/mg protein	2 ± 0.9	2 ± 0.7	0.40
C20-Cer pmol/mg protein	30 ± 11	28 ± 8	0.38
C22-Cer pmol/mg protein	11 ± 6	12 ± 4	0.24
C24-Cer pmol/mg protein	85 ± 51	93 ± 29	0.33
C24:1-Cer pmol/mg protein	23 ± 10	31 ± 9	0.06

Data are shown as means ± SD. Cer = Ceramide

### Correlations between reported dietary intake and adipose tissue inflammation

**Fig. 3:**
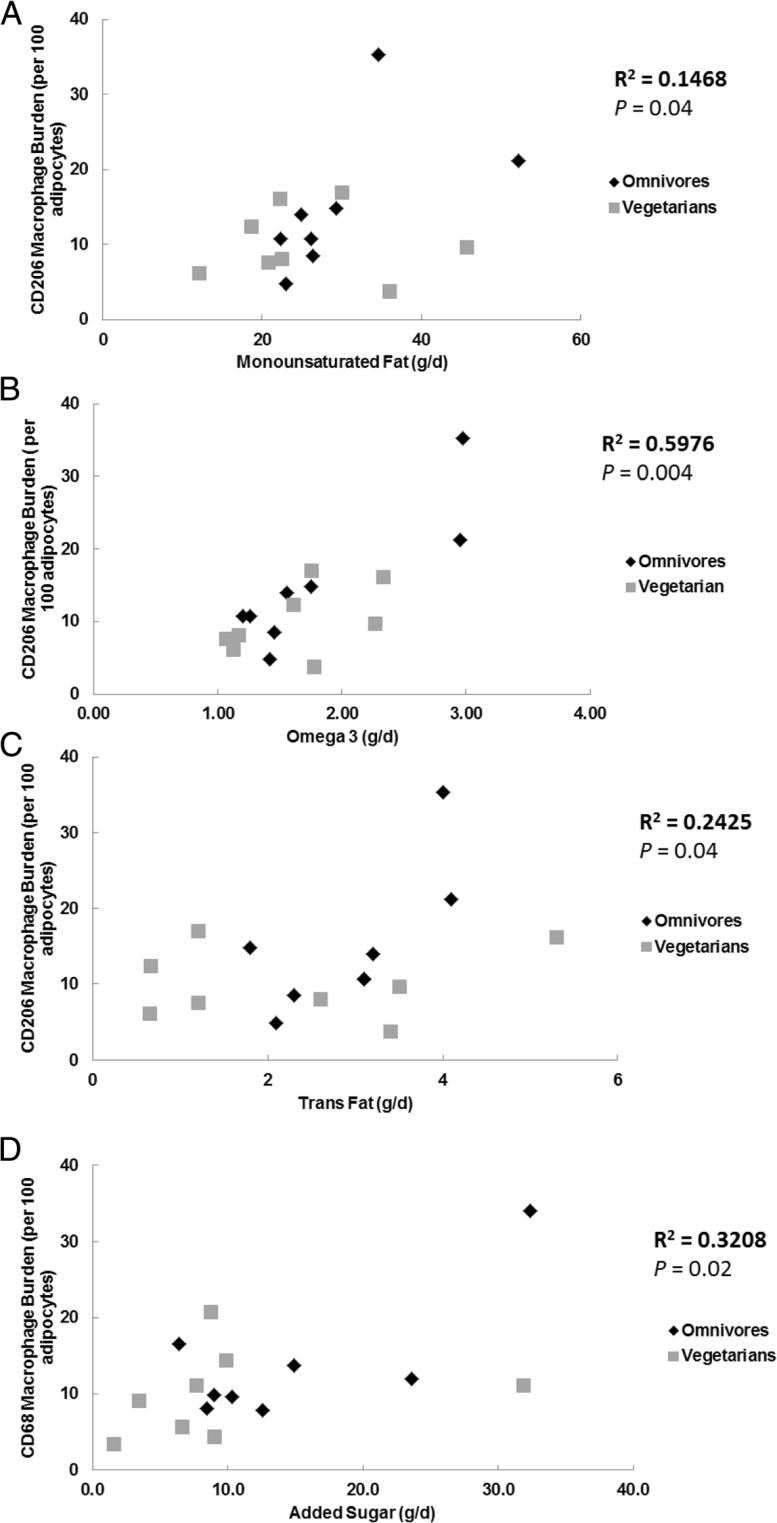
Correlation between abdominal macrophage content quantified via immunohistochemistry (Y-axis) and dietary intake (x-axis). **a**. CD206 positive macrophages and monounsaturated fat intake. **b**. CD206 positive macrophages and omega 3 dietary intake. **c**. CD206 positive macrophages and trans-fat intake. **d**. CD68 positive macrophages and added sugar intake

We found no significant correlation between adipose tissue macrophage burden and reported intake of total calories, total fat, saturated fat or polyunsaturated fat. Following Bonferroni correction, there remained a positive correlation between abdominal CD206 (M2) macrophages and monounsaturated fat intake (*r* = 0.50, *P* = 0.04) as well as omega-3 fatty acid intake (*r* = 0.77, *p* = 0.0004) (Fig. 3a-b). We also found that trans-fat intake was correlated with total macrophage burden (*r* = 0.49, *P* = 0.04), as was added sugar intake (*r* = 0.57, *P* = 0.02) (Fig. 3c-d).

## Discussion

Variations in the quality of the diet are believed to contribute to variations in adipose tissue inflammation [6, 21] and consuming a vegetarian diet is associated with reduced prevalence of a variety of chronic conditions [5]. Although the effect of lipid species on adipose tissue inflammation has been tested in vitro and in animal models, the relationship between a vegetarian diet and human adipose tissue inflammation has not been studied. We assessed whether obese vegetarians have less adipose tissue inflammation than obese omnivores. The vegetarian participants reported consuming significantly less saturated fat and, consistent with this information, had a significantly lesser percentage of plasma FFA palmitate, the most prominent saturated fatty acid. Although muscle bioactive lipid metabolite concentrations were not different between groups, vegetarians had fewer femoral adipose tissue macrophages by some metrics. These data provide some evidence for a beneficial effect of a vegetarian diet on adipose tissue health in obese adults.

The term vegetarian can include food practices that include pescatarian, lacto-, lacto-ovo, and vegan. For the purpose of the current study we included the entire spectrum of vegetarian diets. We chose to study obese omnivores and BMI-matched vegetarians (one vegetarian had a BMI of 28 kg/m^2^) because our database (derived from our methodology studies [16]) indicate that substantial increases in ATMs occur primarily with BMI’s above 30 kg/m^2^. It would be more difficult to detect a difference between omnivores and vegetarians if the omnivores already have a low ATM burden. As expected, we found significant differences in reported dietary intake between the two groups (Table 2). While these differences in diet between the various forms of vegetarianism and omnivores are well established, their association with adipose tissue health has not been studied. It is important to note that, by design, we recruited relatively healthy obese participants (Table 1**)**, which may have made it more difficult to detect differences in inflammation. However, had we not matched the groups for obesity and health status it would have been problematic to address the issue of whether dietary patterns, as opposed to metabolic abnormalities per se, were linked with adipose tissue health.

The lesser saturated fatty acid consumption by vegetarians (Table 2) might explain our findings, because saturated fatty acid exposure is proposed to play a role in systemic, and possibly adipose tissue, inflammation. Previous studies have found that individuals following a vegetarian diet have lower levels of systemic inflammation [6] and that exposure of cultured muscle cells to omega-3 fatty acids can reduce ceramide concentrations [22]. We found that adults following a vegetarian diet for at least 5 years had significantly less expression of abdominal adipose tissue *TNF* mRNA and fewer femoral subcutaneous adipose tissue macrophages per 100 adipocytes. However, we found no significant difference in muscle ceramide content between the omnivore and vegetarian group (Table 6). Previous studies have reported greater muscle insulin sensitivity in vegetarians [23]. Although we did not measure muscle insulin action in our study, if our volunteers were more insulin sensitive than the omnivores it would appear to be independent of muscle ceramides. However, our findings of decreased ATM content in the femoral adipose depot combined with previous studies investigating systemic inflammation suggest that a vegetarian diet may contribute to an anti-inflammatory environment in vivo*.* Indeed, the current study also suggests there is a relationship between adipose macrophage burden and specific dietary factors (Fig. 3**)**. Although we did not observe a significant relationship between saturated fat intake and macrophage burden, we did observe a significant positive correlation between macrophage burden and other known detrimental dietary components such as trans-fat and added sugars. In addition, we found a positive correlation between omega-3 fatty acid intake, proposed to decrease systemic inflammation, and anti-inflammatory CD206 macrophage burden.

In addition, animal models and in vitro models suggest that the stress of obesity and excess intake of saturated fatty acids increase the senescent cell burden in adipose tissue [24]. However, we found no difference in adipose tissue senescent cell burden between the two groups, which may indicate that obesity itself, rather than the diet content, is a more important determinant of senescent cell burden. However, it is important to note that the small sample size increases the risk for a type 2 statistical error. The exact relationship between senescent cell and ATM burden is still unknown and further study is necessary to elucidate this relationship and its clinical significance.

While we found greater abdominal adipose tissue TNF mRNA expression in omnivores, we found no difference between the two groups in the abdominal adipose tissue ATMs. Based on the relationship between abdominal adiposity and chronic disease these findings are not what we would hypothesize. In addition, the omnivore group had significantly greater numbers of CD206/anti-inflammatory (M2) macrophages in the femoral depot than the vegetarian group, another finding that would appear to be contradictory. Further studies assessing the relationship between femoral depot adipose tissue inflammation and cardio-metabolic parameters are necessary to elucidate the significance of these findings. In addition, we found a significant difference between groups when quantifying macrophages per 100 adipocytes but no difference when data was evaluated as per gram tissue. Without further data, it isn’t clear which of these two data expression approaches will be most informative when attempting to understand the relationship between adipose inflammation and cardio-metabolic parameters. Lastly, we found that there were more CD206 macrophages present than total CD68 macrophages in both depots and that the CD14 or M1 macrophage population added to the CD206 or M2 macrophage population is greater than the total CD68 population. This is possible because there are other antigens that can be used to identify the total macrophage population and not all of these macrophages express CD68. Furthermore, macrophages can have antigens for both M1 and M2 macrophages [16]. However, further study to elucidate the link between macrophage burden and disease risk is required.

We observed some interesting differences in the fatty acid composition of the abdominal and femoral adipose tissue. Overall, the femoral depot contained higher proportions of unsaturated fatty acids while the abdominal depot had higher proportions of saturated fatty acids. This is in line with previous studies documenting different proportions of unsaturated fatty acids in different depots [25, 26]. These differences are believed to contribute to the “softer” fat found in the femoral depot but the clinical significance of these differences remains to be elucidated.

There are some limitations to the current study. The sample size (*n* = 8 per group) is small because of the rarity of obesity in vegetarians in our region and in other studies [5], however, we were able to detect significant differences in a variety of our measurements. In addition to the small sample size the vegetarian group was heterogeneous in terms of the spectrum of vegetarian diet, which could make it more difficult to detect differences between omnivores and vegetarians who follow specific dietary patterns.. It is also important to note that the study sample was primarily female, and it is possible that males respond differently. Lastly, only vegetarians that had been following the diet for at least 5 years were included in the current study. Further studies to determine if a shorter duration of the vegetarian can provide similar results are warranted.

## Conclusions

In summary, adults that follow a vegetarian diet for at least 5 years consume less saturated fat intake, which likely accounts for less palmitate in plasms FFA and possibly is related to reduced femoral adipose tissue inflammation. While the relationship between plasma lipid panel values and disease risk are well established, the relationship between ATM burden and chronic diseases remains unknown and further study is necessary.

## Data Availability

The datasets used and/or analysed during the current study are available from the corresponding author on reasonable request.

## Abbreviations

ATM: Adipose tissue macrophage
BMI: Body mass index
Cer: Ceramide
CLS: Crown-like structures
D: Day
DAG: Diacylglycerol
DEXA: Dual-energy X-ray absorptiometry
DHA: Docosahexaenoic acid
EPA: Eicosapentaenoic acid
FFA: Free fatty acid
FFQ: Food frequency questionnaire
G: Gram
IHC: Immunohistochemistry
Kcal: Kilocalorie
LC/MS: Liquid chromatography–mass spectrometry
Mg: Milligram
*n*: Number of subjects
SD: Standard deviation
SEM: Standard error of the mean
